# The Role of Omega-3 Fatty Acids in the Setting of Coronary Artery Disease and COPD: A Review

**DOI:** 10.3390/nu10121864

**Published:** 2018-12-02

**Authors:** Alex Pizzini, Lukas Lunger, Thomas Sonnweber, Guenter Weiss, Ivan Tancevski

**Affiliations:** 1 Department of Internal Medicine II, Infectious Diseases, Pneumology, Rheumatology, Medical University of Innsbruck, 6020 Innsbruck, Austria; alex.pizzini@i-med.ac.at (A.P.); Thomas.Sonnweber@i-med.ac.at (T.S.); Guenter.Weiss@i-med.ac.at (G.W.); 2 Department of Urology, Klinikum rechts der Isar, Technische Universität München, 81675 Munich, Germany; lukas.lunger@tum.de

**Keywords:** omega 3, PUFA, *n*-3 PUFA, COPD, inflammation, coronary artery disease, ischemic heart disease, CAD, CHD

## Abstract

Chronic obstructive pulmonary disease (COPD) is a growing healthcare concern and will represent the third leading cause of death worldwide within the next decade. COPD is the result of a complex interaction between environmental factors, especially cigarette smoking, air pollution, and genetic preconditions, which result in persistent inflammation of the airways. There is growing evidence that the chronic inflammatory state, measurable by increased levels of circulating cytokines, chemokines, and acute phase proteins, may not be confined to the lungs. Cardiovascular disease (CVD) and especially coronary artery disease (CAD) are common comorbidities of COPD, and low-grade systemic inflammation plays a decisive role in its pathogenesis. Omega-3 polyunsaturated fatty acids (*n*-3 PUFAs) exert multiple functions in humans and are crucially involved in limiting and resolving inflammatory processes. *n*-3 PUFAs have been intensively studied for their ability to improve morbidity and mortality in patients with CVD and CAD. This review aims to summarize the current knowledge on the effects of *n*-3 PUFA on inflammation and its impact on CAD in COPD from a clinical perspective.

## 1. Introduction

Chronic obstructive pulmonary disease (COPD) is one of the leading causes of morbidity and mortality worldwide [1]. Data from the Global Burden of Disease Study estimate that, to date, approximately 328 million people suffer from COPD globally, and suggest that this number may be even higher because of a substantial number of undiagnosed cases and increasing exposure to environmental factors, which promote COPD development and progression. It is estimated that, by the year 2030, COPD will represent the third leading cause of death worldwide [1,2,3,4,5].

**Figure 1:**
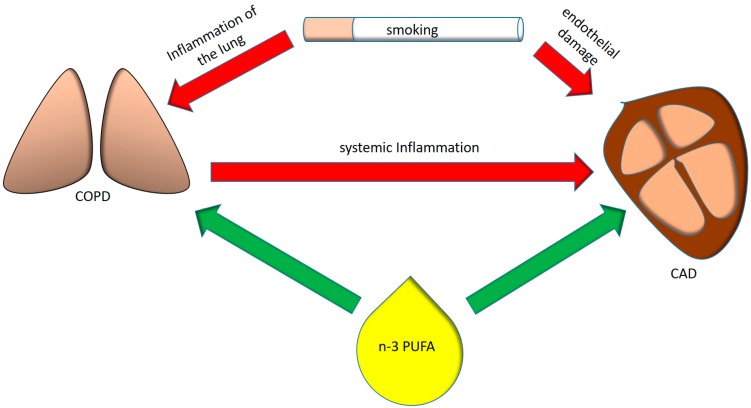
Interplay between smoking, pulmonary and systemic inflammation, coronary artery disease and omega-3 polyunsaturated fatty acids (*n*-3 PUFAs). COPD = chronic obstructive pulmonary disease, CAD = coronary artery disease.

COPD is the result of a complex interaction between lifestyle factors, especially cigarette smoking and air pollution, and genetic preconditions, resulting in persistent inflammation of the airways even after smoking cessation, causing sustained peripheral airflow limitation [1,6]. The latter is defined as the ratio between forced expiratory volume in one second (FEV1) versus forced vital capacity (FVC) being less than 0.7, determined via spirometry after bronchodilator therapy [1]. The exact mechanisms behind these processes are not yet understood, but the challenge to oxidative stress and the resulting imbalance of proteases and anti-proteases seem to be causative [6]. Moreover, there is growing evidence that the chronic inflammatory state reflected by increased levels of circulating cytokines, chemokines, and acute phase proteins may not be confined to the lungs [7]. Cardiovascular disease (CVD) and coronary artery disease (CAD) are the typical and most common comorbidities of COPD, and significantly impact on patients’ prognosis and quality of life (Figure 1) [8,9,10,11].

Omega-3 polyunsaturated fatty acids (*n*-3 PUFAs) exert multiple functions in humans and are crucially involved in limiting and resolving inflammatory processes [12,13]. Eicosapentaenoic (EPA) and docosahexaenoic (DHA) acid are the main long-chain PUFA of the *n*-3 PUFA family, and seafood is the richest dietary source of these essential fatty acids, whereas their metabolic precursor, α-linolenic acid (ALA), is contained in substantial amounts only in vegetables [14].

## 2. Systemic Inflammation in COPD

The principal pathological features of COPD are obstructive bronchiolitis and emphysema, as well as increased mucus secretion. These conditions are the result of a rather complex and chronic inflammatory process, which is initiated mostly by chronic inhalation of irritants, predominantly cigarette smoking, air pollutants, and biomass fuel [6]. In this context, it ought to be mentioned that each puff of a cigarette contains more than 2000 xenobiotic compounds and 1014 free radicals, which injure lung epithelial cells with dose-dependent cytotoxicity [15,16]. These irritants subsequently activate pattern recognition receptors, such as Toll-like receptors (TLR), either directly, or indirectly by causing injury to epithelial cells, which release damage-associated molecular patterns. Interleukin (IL)-1ß has been demonstrated to be crucially involved in this first step, and IL-1 receptor knockout mice exposed to cigarette smoke showed reduced pulmonary inflammation and were significantly protected from development of emphysema compared with littermate controls [17]. On activation, innate immune cells release pro-inflammatory cytokines and chemokines, such as tumor necrosis factor α (TNFα) and IL-8, which leads to further recruitment of neutrophils and inflammatory monocytes to the lung [18]. Activated neutrophils and macrophages, in turn, foster lung destruction through the release of reactive oxygen species and proteolytic enzymes such as neutrophil elastase and matrix metalloproteinases [19,20].

Activation of the adaptive immune system is a second step in the pathogenesis of COPD, and cigarette smoke is crucially involved in activation of dendritic cells in the lower respiratory tract, leading to T cell activation [21]. CD8 cytotoxic T cells are the predominant T cells in the airways of COPD patients [15], however, CD4+ T helper-1 cells and CD4+ T helper-17 cells are also found in high quantities [6,22,23]. Furthermore, B cells organized into lymphoid follicles are frequently detected in the airways of patients with COPD, as shown in central bronchial biopsy specimens [24].

Several inflammatory cytokines, including TNFα; IL-6; IL-8; IL-18; and acute phase proteins such as C-reactive protein (CRP), serum amyloid A, and fibrinogen, as well as white blood cells (WBC), are detected at increased concentrations in the circulation of patients affected by COPD [25,26]. Similarly, these cytokines are also increased in the sputum and bronchoalveolar lavage fluid of COPD patients [8], suggesting a close relationship between airway and systemic inflammation. The latter has been described as an overspill of inflammatory mediators from the peripheral lung to systemic circulation contributing to the development of comorbidities of COPD [7]. Agusti et al. analyzed six inflammatory biomarkers (WBC, CRP, IL-6, IL-8, fibrinogen, and TNFα) in one of the largest available COPD cohorts, the ECLIPSE cohort [26]. The study included COPD patients, smokers without airflow limitation, and non-smokers. Thirty percent of COPD patients had no signs of systemic inflammation, whereas 16% had persistent systemic inflammation. The study group with persistent inflammation had significantly increased all-cause mortality, as compared with individuals without chronic inflammation (13% vs. 2%). The authors concluded that systemic inflammation is not a constant feature in all COPD patients, because approximately one-third of those analyzed did not have any abnormal biomarker neither at baseline nor at one year after follow-up. In a logistic regression analysis, body mass index (BMI), age, current smoking, and airflow limitation were identified as risk factors for persistent inflammation in COPD patients. Similarly, Garcia-Aymerich et al. were able to identify a COPD phenotype characterized by high proportion of obesity, cardiovascular disease, diabetes, and systemic inflammation in approximately one-third of patients recruited at the first hospital admission because of acute exacerbated COPD (AECOPD) [27]. Yet, the mechanisms linking COPD to the development or progression of COPD associated comorbidities are not fully understood, as these studies only show associations and do not prove causality.

## 3. Coronary Artery Disease in COPD

CAD prevalence rates in COPD patients range from 7% to 34% [28,29,30]. COPD and CAD have a close relationship, as they share common risk factors such as advanced age, cigarette smoking, and environmental pollution. There are evident epidemiologic data connecting COPD with CAD. In a cross-sectional study of patients with suspected CAD, patients with COPD had a significantly higher frequency of obstructive coronary lesions as compared with CAD patients without airflow limitation [31]. Additionally, the severity of airflow limitation correlated with coronary lesion size and quantity. No difference was found between COPD and non-COPD CAD patients regarding risk factors, and a univariate analysis revealed COPD as an independent predictor of CAD. Multiple other studies have shown similar results and identified COPD as a powerful and independent risk factor for CVD and CAD. Spirometry tests, especially FEV1, and airflow obstruction defined as FEV1/FVC less than 0.70, for instance, have strong predictive potential for CAD. For every 10% decrease in FEV1, cardiovascular mortality was found to increase by about 28%, and even among individuals with severe airways obstruction, the leading causes of death are predominantly sequels of cardiovascular disease [31,32,33,34]. On the other side, studies investigating airflow limitation in patients with CAD are scarce, but the prevalence of obstructive ventilation in CAD patients might be underestimated in the literature [28].

The pathophysiological mechanism linking COPD to its comorbidities is not yet certain, but chronic exposure to low-grade systemic inflammation might play a central role. Inflammation is a key factor in the development and progression of atherosclerotic lesions, and numerous cytokines, mediators, and immune cells have been identified to promote vascular inflammation, cholesterol deposition in the arterial wall, and the formation of atherosclerotic plaques [35,36].

Most large epidemiologic studies identified C-reactive protein (CRP) as a key player and predictor of CAD [35,37], as well as “upstream” inflammatory cytokines like IL-6, IL-18, and TNFα strongly correlated with disease activity and severity in CAD [38,39]. Similar systemic alterations of systemic inflammatory markers are found in those COPD patients that revealed an increased prevalence of cardiovascular disease [26].

However, even though persistent inflammation may constitute the pathophysiological link between COPD and CAD, alternative mechanistic approaches ought to be mentioned.

Pulmonary emphysema, a phenotype of COPD, is a pathologic condition characterized by abnormal and permanent enlargement of the airspaces distal to the terminal bronchioles that leads to destruction of airspace walls and usually to progressive airflow limitation [1]. Studies about the pathogenesis of emphysema revealed an association with endothelial apoptosis [40,41,42] and endothelial dysfunction [43]. The subsequently altered pulmonary perfusion can lead to impaired left ventricular filling, reduced stroke volume, and lower cardiac output [44]. The endothelial damage, however, is not limited to the lungs and large cross-sectional studies linked the presence of emphysema to subclinical atherosclerosis and peripheral arterial stiffness [45] as a consequence of endothelial and vascular smooth muscle dysfunction [46]. Arterial stiffness is a strong predictor of cardiovascular disease [47,48], and emphysema severity based on quantitative computed tomography scans in turn was revealed as the most powerful predictor of arterial stiffness [49]. Moreover, systemic vascular dysfunction seems to already be present in the earlier stages of COPD, and particularly in patients with emphysema, although presenting with a largely preserved FEV1 [50].

Still, many other potential mechanisms, for instance, physical inactivity secondary to more advanced COPD stages, chronic hypoxia, genetic predisposition, as well as direct endothelial damage caused by cigarette smoking, may help to further explain the frequent association between COPD and CAD [51].

## 4. Anti-Inflammatory Proprieties of *n*-3 PUFA and the Benefits in Cardiovascular Disease

*n*-3 PUFA exert multiple functions in humans and are crucially involved in limiting and resolving inflammatory processes. A recent American Heart Association science advisory recommends consuming nonfried seafood, especially species higher in long chain *n*-3 PUFA, one to two times per week for cardiovascular benefits [52]. However, data about the specific *n*-3 PUFA supplementation for influencing the risk of CVD are still ambiguous, underlined by recent findings from randomized controlled trials and meta-analyses, which led to a class III recommendation by the American Heart Association for dietary supplementation with *n*-3 PUFAs in populations at high risk of CVD [53]. This is further supported by a Cochrane Systematic Review providing information that EPA and DHA slightly reduce serum triglycerides and raise high density lipoprotein cholesterol (HDL-C), however, little or no effect on all-cause deaths and cardiovascular events and probably little or no difference in terms of cardiovascular death, coronary deaths or events, stroke, or heart irregularities was described. [54]. The hypothesized protective mechanisms are not solely related to reduced overall inflammation, but also include effects on lipid metabolism and thrombogenesis, as well as anti-arrhythmic proprieties, reducing the rate of sudden cardiac death in secondary prevention [53,55,56].

Increased bioavailability of *n*-3 PUFAs, by means of EPA and DHA, change the balance between *n*-3 and *n*-6 PUFA with arachidonic acid (AA) as the major precursor of the latter, favoring the synthesis of anti-inflammatory eicosanoids [57]. The incorporation of EPA and DHA into human inflammatory cells occurs in a dose-response fashion, and because less substrate is available for synthesis of AA-derived eicosanoids, *n*-3 PUFA supplementation to human diet has been shown to result in decreased production of prostaglandin E2 (PGE2), thromboxane A2, Leukotriene B4 (LTB4), 5-hydroxyeicosatetraenoic acid, and leukotriene E4 by inflammatory cells [57]. Plenty of evidence supporting this hypothesis is available from rheumatoid arthritis disease, a disease primarily characterized by inflammation [58]. There are many studies about patients with rheumatoid arthritis, where fish oil supplements decreased LTB4 production by neutrophils and monocytes, 5-hydroxyeicosatetraenoic acid production by neutrophils, and PGE2 production by mononuclear cells [58]. A recently published prospective trial concluded that the intake of fish oil resulted in a shift of *n*-6/*n*-3 PUFA ratio and in a higher incorporation of *n*-3 PUFA precursors for the anti-inflammatory lipid mediators in plasma phospholipids. This further induced a significant improvement in the clinical status by improving joint inflammation, as well as reducing inflammatory parameters (CRP, erythrocyte sedimentation rate) [59]. However, the altered ratio between *n*-6/*n*-3 PUFA occurred solely through an increase of *n*-3 PUFA, while *n*-6 PUFA remained unchanged. In the literature, similar concerns with the *n*-6/*n*-3 PUFA ratio were identified and discussed. Depending on how the proportion of *n*-6 PUFA changes, the proportion of *n*-3 PUFA could decrease, remain unchanged, or increase and still lead to a lower *n*-6/*n*-3 PUFA ratio, although the active biochemical effects of a decrease, no change, or an increase in the proportion of *n*-3 PUFA are distinctly different. Moreover, the single aspect of an *n*-3/*n*-6 PUFA ratio neglects the source of *n*-3 PUFA, and it is well established that the biological effects of α-linolenic acid (ALA), EPA, and DHA are diverse [60]. In terms of cardiovascular diseases, one would hypothesize that higher levels of *n*-6 PUFA and lower levels of *n*-3 PUFA could increase cardiovascular risk. This has not been demonstrated, as both *n*-6 and *n*-3 fatty acids are associated with reduced cardiovascular risk in several studies [61]. Similarly, two prospective trials concluded that the ratio of *n*-6/*n*-3 polyunsaturated fatty acids is of no value in modifying cardiovascular disease risk [62,63,64].

**Figure 2:**
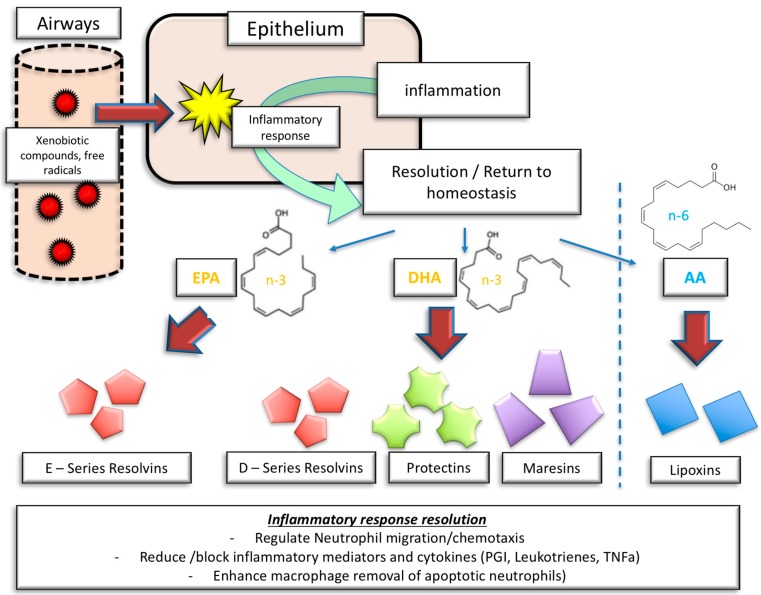
Inflammatory response and inflammatory resolution: the role of molecular mediators derived from *n*-3 PUFA and *n*-6 PUFA. EPA = eicosapentaenoic acid, DHA = docosahexaenoic acid, AA = arachidonic acid, PGI = prostaglandins, TNFα = tumor necrosis factor α.

The anti-inflammatory mechanism of *n*-3 PUFA also expands on leukocyte recruitment by affecting the expression of adhesion molecules on endothelial cells [65]. This step is crucial, as monocytes in particular migrate into the vessel wall, differentiate into macrophages, and may become foam cells, contributing to the initiation and progression of inflammatory atherosclerotic lesions [66,67]. Moreover, resolution of inflammation is an actively programmed biochemical progress, regulated by specialized pro-resolving mediators (SPM) derived from *n*-3 and *n*-6 PUFA (Figure 2) [68]. While *n*-3 PUFAs are synthesized to resolvins, protectins, and maresins, *n*-6 PUFAs are synthesized to lipoxins [69]. SPMs are predominantly involved in the regulation of neutrophil activity, as well as cyto- and chemokine release, and induce the resolution of an inflammatory tissue state towards tissue homeostasis [70].

Figure 2 illustrates the complexity of SPM involvement in inflammation resolution. Resolvin E1, derived from EPA, is involved in the limitation of neutrophil migration, the enhancement of neutrophil and eosinophil clearance, and the reduction of IL-23 and 12 release. Also, resolvin E1 inhibits platelet and vascular smooth muscle activation [71].

Similarly, resolvin D1, derived from DHA, limits neutrophil transmigration, and promotes the clearance of apoptotic cells and allergens. Moreover, resolvin D1 stimulates the production of IgM and IgG. Resolvin D2, on top of limiting neutrophil activity and enhancing macrophage activity, reduces the possibility of leukocyte–endothelial interaction by stimulating NO production [71].

Protectin D1 effectively inhibits T cell migration and apoptosis, and reduces the production and release of TNFα and Interferon gamma, besides limiting neutrophil activity and promoting macrophage activation [70].

Maresin 1, on the other hand, predominately affects pulmonary tissue by reducing the overall amount of neutrophils in the lung, limiting edema, tissue hypoxia, and pro-inflammatory cytokines [70]. Also, IL-5 and IL-13 secretion is reduced, thereby affecting innate lymphoid cells. Another beneficial effect of this SPM is the stimulation of regulatory T cells, stimulating innate lymphoid cells type 2 mediated effects [70]. Finally, *n*-3 PUFAs also directly impact arterial stiffness by decreasing the pulse wave velocity and improving arterial compliance [72], as was shown in a meta-analysis by Pase et al. Similarly, the effects were further confirmed by more recent randomized controlled trials in patients with metabolic syndrome and patients at elevated cardiovascular risk [73,74].

## 5. *n*-3 PUFA in COPD Patients, Smokers, and Subjects at Risk

The beneficial effects of an inflammation-modulating diet using *n*-3 PUFA in patients with acute lung injury or acute respiratory distress syndrome have been reported previously [75,76]. Therefore, it was hypothesized that *n*-3 PUFA might also have beneficial effects in patients with COPD and the effect might extend to linked comorbidities potentially related to systemic inflammation, including CAD.

Investigations on *n*-3 PUFA and the impact on airflow limitation and smoking were published in the past (a schematic overview of the discussed studies is given in Table 1). One of the first available studies was conducted by Sharp et al. in 1994 [77]. A large cross-sectional cohort study (Honolulu Heart Program) revealed a protective function of elevated fish consumption (major source of *n*-3 PUFA) in smokers by decelerating the FEV1 decline. However, the effect was limited to subjects with a maximum inhalation of 30 cigarettes per day, indicating a dose-dependent limitation. Interestingly, an analysis of the same cohort also showed a decrease in the risk of CAD morbidity and mortality in smokers with increased fish intake (defined as >2 times/week), suggesting a relationship between airflow limitation, CAD, and *n*-3 PUFA [78]. No information is available on whether patients matching the current definition of COPD were included in this analysis.

Shahar et al. analyzed the dietary impact of *n*-3 PUFA (assessment of EPA and DHA intake via questionnaire) on 8960 current or former smokers from the “Atherosclerosis Risk in Communities—ARIC” study cohort and studied its relation with COPD [79]. COPD was assessed by a questionnaire on respiratory symptoms and by spirometry. The definition of COPD, however, was slightly different when compared with the current GOLD definition [1]. The authors describe a strong inverse relation between COPD severity and intake of *n*-3 PUFA. Moreover, higher intake of *n*-3 PUFA and fish consumption also predicted better lung function in smokers and former smokers. Many other investigations, including the same cohort, were subsequently published; *n*-3 PUFAs were associated with a modification of hemostatic factors—key components of CAD and acute myocardial infarction—resulting in a hypocoagulable profile [80]. Data from the same cohort also revealed lower levels of plasma PUFA (especially *n*-6 PUFA) among participants who developed CAD [81]. Yamagishi et al. found an increased risk of heart failure in subjects with higher plasma levels of saturated fatty acids, and *n*-3 PUFAs were associated with a decreased risk of heart failure in women in the ARIC cohort [82].

A positive association between *n*-3 PUFA (assessment of *n*-3 PUFA via questionnaire) consumption and ventilatory function (FEV1% and FVC%) was found in 1232 adults aged between 22 and 28 years [83]. The analyzed cohort had no signs of obstructive ventilatory dysfunction, and statistical significance was lost after adjusting for multiple comparisons, suggesting only a weak effect of *n*-3 PUFA. The idea of analyzing a cohort of young adults was related to the fact that reduced maximal attained lung function (as measured by spirometry) may identify individuals who are at increased risk for the subsequent development of COPD [1,84].

Leng et al. conducted a nutritional epidemiological study in two separate cohorts (Hispanic and non-Hispanic white smokers; assessment of EPA, DHA, and docosapentaenoic acid (DPA) intake via questionnaire) to identify nutrients associated with FEV1 and its decline [85]. Of the analyzed *n*-3 PUFAs, EPA, DHA, and DPA intake were positively associated with better FEV1 volumes, and DPA was associated with a reduced age-related FEV1 decline. No increased FEV1 decline of currently smoking participants was observed in one of the two cohorts (Lovelace Smoker’s cohort) if high amounts of DPA were consumed. The study only included approximately 25% COPD patients, and both cohorts showed a mean FEV1/FVC ratio of >0.7, limiting the disease to mild-to-moderate stages. No data about CVD prevalence in the two analyzed cohorts are provided.

Conflicting evidence came from another population-based cohort study showing that an increased proportion of dietary fat consumption was associated with increased IL-6 levels, which were significant predictors of FEV1 and FVC [86]. Plasma IL-6 is a clinically relevant marker of inflammation and has been linked to cardiovascular outcomes in some epidemiological studies [87]. *n*-3 PUFA (assessment via questionnaire) intake was not found to be associated with %FEV1 in the linear regression model, and no information is given about the relation between *n*-3 PUFA and IL-6. Overall, the patients included in the study had no absolute limitation of FEV1 (approximately 80% predicted) and obstructive ventilation, a typical feature of COPD, was not found (FEV1/FVC > 0.7.) Moreover, the assessed smoking status revealed non-smokers in 73% of female patients and 39% of male patients and, therefore, the odds of COPD patients being included in the study are low.

In 2016, Scaglia et al. analyzed dietary habits related to PUFA consumption (red blood cell membrane determination of alpha linoleic acid (ALA), EPA, DPA, and DHA) in smokers and lifetime non-smokers [88]. They found significant differences in the dietary consumption of *n*-3 PUFA between the two groups and showed lower levels of DHA and EPA in smokers. They hypothesized that PUFA might interfere with smoking habits, suggesting dietary supplementation as a potential therapeutic option for smoking cessation. Still, this observation was not confirmed in follow up studies thus far and urges further elucidation.

The anti-inflammatory proprieties of *n*-3 PUFA in COPD patients have been studied [89] in sixty-four patients receiving either an *n*-3 PUFA (ALA) or an *n*-6 PUFA (linoleic acid) supplementation diet for two years. Apart from clinical improvements (decrease of dyspnea according to Borg scale and decrease of arterial oxygen saturation after a 6 min walking test), leukotriene B4 levels in serum and sputum, as well as TNFα and IL-8 levels in sputum, decreased significantly in patients consuming an *n*-3 PUFA rich diet, while there was no significant change in individuals receiving dietary supplementation of *n*-6 PUFA.

A cross-sectional study including 250 COPD patients analyzed inflammatory parameters related to dietary intake of *n*-3 (EPA, DHA, ALA) and *n*-6 PUFA [90]. Higher intake of alpha linoleic acid (ALA) was associated with lower TNFα concentrations in serum, while no differences were seen with other assessed inflammatory parameters (IL-6, IL-8, and CRP). A higher ALA intake was related to higher IL-6 and CRP serum concentrations.

The effects of PUFA supplementation (blend of PUFA, 9 g, mainly *n*-3 PUFA, namely ALA, EPA, and DHA) on the outcome of pulmonary rehabilitation were studied in 80 COPD patients randomized to an intervention or a placebo group [91]. The peak exercise capacity increased in both groups. However, a significantly higher increase was observed in the intervention group after eight weeks of training. Also, the duration of the constant work rate increased far more in patients receiving PUFA. However, the measured systemic inflammatory parameters, namely CRP, IL-6, and TNFα, did not change after rehabilitation or after PUFA intervention, contrasting the results of others [89,90], who observed modulation of systemic inflammation after *n*-3 PUFA supplementation. Thus, systemic inflammation is not a common feature of all COPD patients. Therefore, a more detailed analysis of the subgroup of “inflamed COPD” patients would be desirable. Still, it is unanswered as to how the biochemical phenotype of an “inflamed COPD” patient is defined.

The beneficial effects of *n*-3 PUFA in COPD associated cachexia were studied in a randomized controlled trial including 45 patients [92]. Patients in the intervention group received 2.0 g DHA + EPA as well as 10 g whey protein concentrate and 10 μg 25-hydroxy-vitamin D3 twice daily for 12 weeks versus a milk-based comparator. Both groups gained weight, but the intervention group gained more fat mass. Moreover, a significant reduction in systolic blood pressure and increases in high-density lipoprotein cholesterol were observed in the intervention group compared with controls, parameters that can be regarded as risk factors for CAD [93]. No significant changes in the measured inflammatory parameters (CRP, IL-6, IL-8, and TNF) were observed.

In summary, several limitations of the described studies must be discussed. First, the most important limitation is that most of the studies are cross-sectional observational cohort studies. Second, *n*-3 PUFA sources are indirectly measured using food frequency questionnaires. Dietary habits reported from questionnaires are surrogate parameters of the real dietary consumption of *n*-3 PUFA; this may lead to inaccurate *n*-3 PUFA consumption estimations, although studies suggest a plausible correlation between the two factors [94,95]. Third, the causality and the direction of a causal relation cannot be explained when considering a cross-sectional study design. Finally, COPD patients and smokers may have changed their lifestyles and dietary habits, reducing fish consumption. Therefore, further prospective studies are warranted to investigate the impact of *n*-3 PUFA consumption on systemic inflammatory parameters in such patients.

## 6. *n*-3 PUFA, CAD, and COPD

Evidence on the beneficial effects of *n*-3 PUFA on CAD and CVD in COPD patients is scarce. Many large randomized controlled trials have been conducted to study the benefits of *n*-3 PUFA in CAD and CVD [53,54], however, although most of the included patients were smokers, the prevalence and severity of COPD were rarely reported.

We reviewed all included studies from the Cochrane systematic database review, “Polyunsaturated fatty acids for the primary and secondary prevention of cardiovascular disease”, and the American Heart Association Science Advisory, “Omega-3 Polyunsaturated Fatty Acid (Fish Oil) Supplementation and the Prevention of Clinical Cardiovascular Disease”, in regard to included COPD patients [53,54]. Of the included 65 studies, only 4 reported on COPD patients in their cohort.

Nodari et al. analyzed the benefits of *n*-3 PUFA (850 to 882 mg of EPA and DHA) in the prevention of atrial fibrillation (AF) recurrence rates after electrical cardioversion in a prospective randomized controlled trial, including 199 patients [96]. After one year of follow-up, the probability of maintained sinus rhythm was significantly higher in the *n*-3 PUFAs-treated patients as compared with the placebo group. A small subgroup of approximately 10% COPD patients (*n* = 12 placebo vs. *n* = 13 intervention) was included in the study, however, no separate details on the subgroup were discussed.

Rauch et al. analyzed the effect of *n*-3 PUFA (460 mg EPA, 380 mg DHA) if given on top of modern guideline-adjusted therapy in survivors of acute myocardial infarction [97]. The study was designed as a randomized, placebo-controlled trial with a duration of one year, and included 3851 patients, of whom approximately 6% were highlighted as COPD patients (*n* = 122 placebo vs. *n* = 108 intervention group). Overall, guideline-adjusted treatment of acute myocardial infarction already resulted in a low rate of sudden cardiac death and other clinical events within one year of follow-up, and the application of *n*-3 PUFA could not further improve the outcome. No details are provided regarding such effects in the COPD subgroup.

The GISSI-HF investigators published a randomized, double-blind, placebo-controlled trial analyzing the effect of *n*-3 PUFA (850–882 mg EPA and DHA) in patients with chronic heart failure [98]. In total, 6975 patients with chronic heart failure of New York Heart Association class II–IV were randomly assigned to *n*-3 PUFA intervention or placebo. A subpopulation of 205 COPD patients was included in the study (*n* = 793 placebo vs. *n* = 740 intervention). Primary endpoints were time to death from any cause or death/admission to hospital for a cardiovascular reason. A slight, yet significant reduction of death from any cause or death/admission to hospital for cardiovascular reasons was found in the PUFA group. No specific details are provided for the COPD subpopulation.

Finally, Mozaffarin et al. analyzed the effect of *n*-3 PUFA (EPA 465 mg and DHA 375 mg) in the prevention of AF in 1516 patients who underwent cardiac surgery [99]. Patients were randomized to receive fish oil or placebo until hospital discharge or postoperative day 10. A small subgroup of approximately 11% of COPD patients (placebo *n* = 90 vs. intervention *n* = 80) was included in the study. The authors concluded that perioperative supplementation with *n*-3 PUFA did not reduce the risk of postoperative AF and, similarly to the aforementioned studies, no details are provided about the COPD subgroup.

In summary, these studies showed a weak benefit of *n*-3 PUFA supplementation in the setting of atrial fibrillation recurrence rate after electrical cardioversion and a slight reduction of death and hospital admission in patients with left heart failure, both diseases closely associated with CAD. No benefits were seen in survivors of acute myocardial infarction or patients with atrial fibrillation after cardiac surgery. The weak benefits seem to also apply to COPD patients, which were included in 5–20% of the discussed studies. However, as already mentioned earlier, the high number of undiagnosed COPD cases and low awareness of investigators towards this chronic disease in patients with cardiac pathologies led to neglect of this important aspect even in large and prospective trials. In our opinion, it seems plausible that other studies also included COPD patients at a rate of at least 5–10%. Thus, the results from the Cochrane systematic database review and the American Heart Association Science Advisory might also apply to COPD patients.

## 7. Conclusions and Future Perspectives

COPD represents a growing healthcare concern worldwide. Pathophysiological aspects of COPD imply a local inflammatory reaction, initiated by inhalation of noxious substances, which, in some cases, led to the emergence of inflammatory mediators in the systemic circulation. Epidemiologic and mechanistic studies indicate that COPD is frequently associated with CAD, congestive heart failure, and cardiac arrhythmias, independent of shared risk factors. A possible pathway is highlighted as chronic low-grade systemic inflammation, an aspect that likely also contributes to coronary artery disease. *n*-3 PUFAs have been intensively studied for their ability to improve morbidity and mortality in patients with CAD. However, little evidence exists toward the beneficial anti-inflammatory aspects of *n*-3 PUFA in COPD patients, and even less evidence is available in regard to patients with COPD and associated CAD.

Overall, the available literature suggests a weak benefit of *n*-3 PUFA in COPD patients related to clinical outcomes, and *n*-3 PUFA mediated borderline amelioration of local and systemic inflammation. To date, the specific interplay between *n*-3 PUFA, COPD, and CAD has not been investigated in prospective trials.

In-depth keyword guided research on ongoing trials on *n*-3 PUFA in COPD and CAD revealed a relatively small number of registered trials on http://ClinicalTrials.gov. To date, only one trial investigating the effect of fish oil on endothelial function by FMD (flow-mediated dilation) in COPD has been registered (NCT00835289). Another trial has investigated the effect of both training and nutritional supplementation on body composition, activity, and cardiometabolic risk in COPD (NCT01344135). However, as nutritional supplementation has not occurred in all subjects and as an observational period of eight months may not be enough to reach clinically important cardiovascular and COPD endpoints, prospective, randomized controlled studies are warranted. Future studies ought to measure atherosclerotic disease progression, as well as establish relevant COPD inflammation parameters in order to determine the role of PUFA in inflammation-mediated diseases such as COPD and CAD. Moreover, standardized PUFA administration as well as a sufficient observation period may be crucial to understanding the role of PUFA in these diseases. Successful trials may help introduce a standardized supplementation of PUFA in patients with CAD or COPD or both to reduce disease progression or development.

## Figures and Tables

**Table 1:** Summary of the reviewed studies related to omega-3 polyunsaturated fatty acid (*n*-3 PUFA) in chronic obstructive pulmonary disease (COPD) patients, smokers and subjects at risk. ALA = alpha linoleic acid, CAD = coronary artery diseases, CRP = C-reactive protein, CVD = cardiovascular disease, FFQ = food frequency questionnaire, DHA = docosahexaenoic acid, EPA = eicosapentaenoic acid, FEV1 = forced expiratory volume in one second, FVC = forced vital capacity, HF = heart failure, IL = interleukin, RCT = randomized controlled trial, STA = stearinic acid, TNFα = tumor necrosis factor alpha.

Author/Year/Type	Cohort Size/Description	*n*-3 PUFA Source	Outcome	COPD	Limitations	Association with CAD (Same Study or Same Cohort)
Sharp et al., 1994, observational	*n* = 6346, Honolulu Heart Program—mean age 54 years	Fish consumption/week—FFQ	Slower decline of FEV1 in smokers.	Obstructive lung disease was solely based on FEV1<65%. Symptoms are not reported.	No details provided about COPD patients. PUFA source reported as fish consumption per week.	Decrease in the risk of coronary heart disease morbidity and mortality in smokers with elevated fish intake.
Shahar et al., 1994, observational	*n* = 8960, Atherosclerosis Risk in Communities Study (ARIC); age: 45–64 years	*n*-3 PUFA (EPA and DHA)—FFQ	Strong inverse relation between COPD and intake of *n*-3 PUFA. Besides COPD, better lung function also in smokers and former smokers.	COPD patients included, however, using a different definition.	Outdated definition of COPD.	*n*-3 PUFAs were associated with hypocoagulable hemostasis. Lower levels of plasma PUFA (especially *n*-6 PUFA) among participants who developed coronary heart disease. Increased risk of HF in subjects with higher plasma levels of saturated fatty acids. *n*-3 PUFAs were associated with a decreased risk of HF in women.
Garcia-Larsen et al., 2015, cross-sectional	*n* = 1232, age 22–28 y	*n*-3 PUFA—FFQ	Positive association between *n*-3 PUFA consumption and ventilatory function (FEV1% and FVC%).	-	Results were not significant after adjustment for multiple comparisons.	-
Leng et al., 2017, observational	*n* = 1829, Lovelace Smokers cohort and *n* = 508 the Veterans Smokers cohort, age 40–74 years	*n*-3 PUFA, EPA, DPA, DHA—FFQ	EPA, DPA, and DHA were associated with better FEV1; DPA was associated with a lower age-related FEV1 decline.	25% COPD patients.	Both cohorts showed a mean FEV1/FVC ratio of >0.7. Included COPD patients mild-to-moderate stages.	-
Wood et al., 2010, observational	*n* = 195, Hunter community study, age 55–85 years	*n*-3 PUFA—FFQ	Increased proportion of dietary fat was associated with increased IL-6 levels, which were significant predictors of FEV1% and FVC in men. No association between *n*3-PUFA and %FEV1.	No information given (no absolute limitation of FEV1% and FEV1/FVC > 0.7).	No information is given about the relation between *n*-3 PUFA and IL-6.	IL-6 is an important marker of cardiovascular and coronary artery disease. No data about CAD or CVD reported.
Scaglia et al., 2016, cross-sectional	*n* = 100 (1:1 smokers vs. never-smokers), 18–75 years	Red blood cell membrane determination of ALA, EPA, DPA, and DHA	Significantly lower levels of DHA in smokers compared with non-smokers.	-	Spirometry not included; no information about lung diseases.	-
Matsuyama et al., 2005, prospective interventional, 2 years duration	*n* = 64, mean age 66 years.	Supplementation *n*-3 PUFA vs. *n*-6 PUFA	Decrease of Borg dyspnea scale and a decrease of arterial oxygen saturation after 6 min walking test in *n*-3 PUFA group. Leukotriene B4 levels in serum and sputum and tumor necrosis factor alpha and interleukin-8 levels in sputum decreased significantly in the *n*-3 group. No change in FEV1.	Limited to COPD only with FEV1% < 60% and BMI <25 kg/m², no active smokers.	Sample size	Impact on inflammatory parameters related to CAD and CVD, however, no data about CAD and CVD in this population are reported.
De Batlle, 2012, cross-sectional	*n* = 250, PAC-COPD study group, mean age 68 years	*n*-3 PUFA: DHA, EPA, ALA—FFQ	ALA was associated with lower TNFα concentrations in serum. AA (*n*-6 PUFA) was related to higher IL-6 and CRP serum concentrations.	COPD patients recruited during their first hospital admission. Mostly moderate to severe COPD.	No repeated measurements, inflammatory sputum parameters not assessed.	Association between ALA and cardiovascular disease, however, no information about CVD or CAD in this population are reported.
Broekhuizen et al., 2005, prospective interventional double-blind RCT over 8 weeks (PUFA vs. Placebo)	*n* = 80, mean age 63 years	9 g PUFA (STA, ALA, EPA, DHA) or placebo daily	Increase in peak exercise capacity and duration of constant work rate. No changes in terms of CRP, IL-6 and TNFa	Mostly severe to very severe COPD patients with mean FEV1% 35–38%.	Limited observation period of 8 weeks.	Association between the analyzed inflammatory parameters and CAD and CVD, however, no information about CVD or CAD in this population are reported.
Calder et al., 2018, prospective interventional RCT over 12 weeks	*n* = 45, mean age 69 years	2.0 g DHA + EPA, 10 g whey protein concentrate and 10 μg 25-hydroxy-vitamin D3 twice daily for 12 weeks vs. a milk-based comparator	Intervention group gained more fat mass. Reductions in systolic blood pressure, triglycerides and exercise-induced fatigue and dyspnoea, and increases in high-density lipoprotein cholesterol.	Moderate-to-severe COPD and involuntary weight loss or low body mass index (16–18 kg/m^2^).	Sample size	Inclusion of metabolic parameters and inflammatory parameters clearly associated with CVD and CAD.
